# Supplementary material for: Reversible Thiol Oxidation Increases Mitochondrial Electron Transport Complex Enzyme Activity but Not Respiration in Cardiomyocytes from Patients with End-Stage Heart Failure
Source: Cells. 2022 Jul 25;11(15):2292. doi: 10.3390/cells11152292 (PMC9330889; doi:10.3390/cells11152292)
Supplement: Supplementary file 1 [file cells-11-02292-s001.zip › cells-1771845-supplementary.pdf]

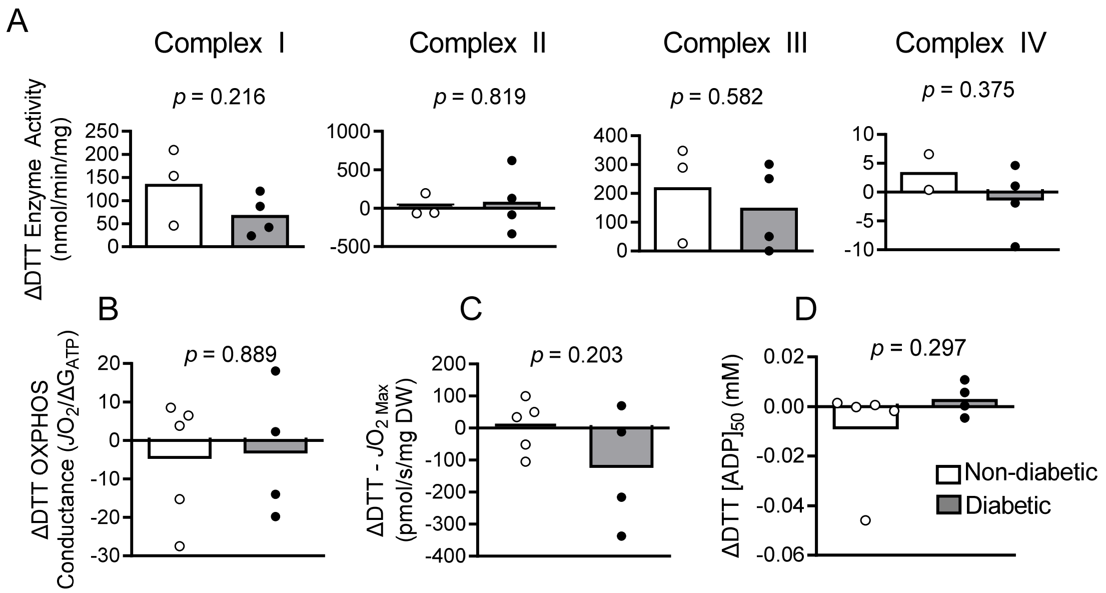

Figure S1. Comparisons between patients with and without diabetes mellitus. ΔDTT (DTT – untreated samples) for mitochondrial parameters assessed in isolated mitochondria (**A**); and permeabilized cardiomyocyte bundles (**B**–**D**). There were no observable differences for the effects of DTT between patients with and without diabetes for (**A**) mitochondrial ETS enzyme activities, (**B**) OXPHOS Conductance, (**C**) maximal respiration, or (**D**) ADP sensitivity. Groups compared made via independent *t*-test.
